# Challenging anticoagulation decisions in atrial fibrillation: a narrative review

**DOI:** 10.1177/17539447241290429

**Published:** 2024-10-16

**Authors:** Michael Griffin, Riccardo Proietti, Gregory Y. H. Lip, Azmil H. Abdul-Rahim

**Affiliations:** Liverpool Centre for Cardiovascular Science at University of Liverpool, Liverpool John Moores University and Liverpool Heart and Chest Hospital, Thomas Drive, Liverpool L14 3PE, UK; Liverpool Centre for Cardiovascular Science at University of Liverpool, Liverpool John Moores University and Liverpool Heart and Chest Hospital, Liverpool, UK; Liverpool Centre for Cardiovascular Science at University of Liverpool, Liverpool John Moores University and Liverpool Heart and Chest Hospital, Liverpool, UK; Danish Center for Health Services Research, Department of Clinical Medicine, Aalborg University, Aalborg, Denmark; Liverpool Centre for Cardiovascular Science at University of Liverpool, Liverpool John Moores University and Liverpool Heart and Chest Hospital, Liverpool, UK; Stroke Division, Department of Medicine for Older People, Whiston Hospital, Mersey and West Lancashire Teaching Hospitals NHS Trust, Prescot, UK

**Keywords:** atrial fibrillation, atrial high-rate episodes, oral anticoagulation, sepsis, stroke, surgery

## Abstract

Atrial fibrillation (AF) is common and warrants consideration of oral anticoagulant (OAC) medication. Usually, the decision is straightforward, following the pathway outlined in the European Society of Cardiology’s guideline; however, certain situations fall outside of this evidence base – such as a diagnosis of subclinical AF made via implanted devices or wearable electrocardiogram monitors, or alternatively diagnosis of ‘secondary AF’ following a major stressor. Subclinical AF is associated with stroke, though not to the extent of clinical AF, and the benefits of anticoagulation appear to be lower. Longer episodes are more clinically meaningful, and recent randomised controlled trials have demonstrated that some patients derive benefit from OAC. Similarly, when AF is triggered by sepsis or non-cardiac surgery, specific evidence supporting OAC initiation is lacking and clinician behaviour is variable. Observational data demonstrate poorer outcomes in these patients, implying that the perception of a transient, reversible phenomenon may not be correct. Contrastingly, cardiac surgery very frequently induces AF, and the benefits of anticoagulation rarely outweigh the risks of bleeding. Following ischaemic stroke, recent evidence suggests that early (re-)initiation of OAC should be considered as this does not increase the risk of haemorrhagic transformation as previously hypothesised. This narrative review summarises the available literature and outlines, where possible, practical advice for clinicians facing these common clinical dilemmas.

## Introduction

Atrial fibrillation (AF) is the most prevalent sustained cardiac arrhythmia, with a lifetime risk of approximately one in three.^1^ AF is strongly associated with ischaemic stroke such that stroke prevention with anticoagulation is at the core of current AF guidelines.^2,3^ Oral anticoagulant therapy (OAC) is indicated in patients with AF and a CHA_2_DS_2_-VASc score of ⩾1 in men and ⩾2 in women. Generally, decision-making to initiate anticoagulation or not is straightforward, but some clinical situations exist for which the evidence in favour of OAC is less clear-cut and real-world practice is more variable.

Common reasons for such clinical dilemmas often fall into one (or more) of three groups. First, the diagnosis of AF may be based on recordings of the heart’s electrical activity from sources other than a standard 12-lead electrocardiogram (ECG). Examples may include pacemaker devices, implantable cardiac monitors, or more recently, commercial wearable technology such as smartwatches.

Second, a diagnosis of new-onset AF may have been made in the presence of a significant physiologic stressor, such as sepsis, critical illness, or cardiac or non-cardiac surgery. If a patient reverts to sinus rhythm with treatment of the underlying stressor and no further episodes of AF are observed, there may be uncertainty regarding the long-term risks of recurrence and stroke.

Third, the benefits of anticoagulation may be felt to roughly balance with the risks of bleeding, for example, in patients with a history of a major bleeding event.

All three scenarios involve difficulties in accurately predicting stroke and/or bleeding risk in very diverse populations. Despite decades of research interest in AF, many challenges persist in relating data to individual patients.

Of these groups, the third is the most clinically variable and complex, as such generalisations may be less applicable, and a highly individualised approach is required. Hence, this review will focus on the first and second groups, providing a summary of the available evidence in a fast-moving field. In so doing, we aim to offer clinicians practical approaches to these challenging scenarios.

**Figure 1.**
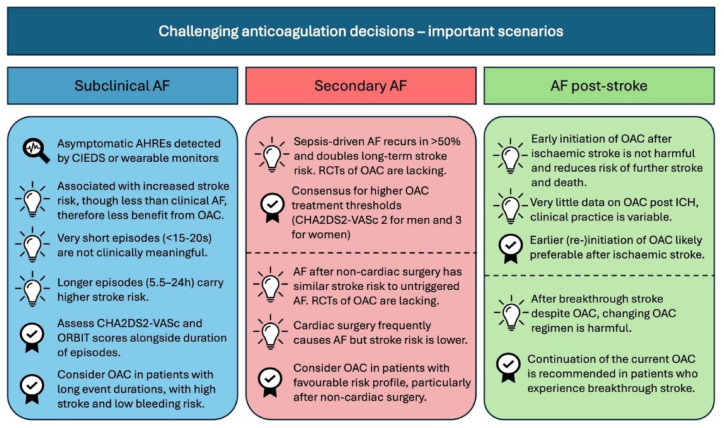
A summary of the key messages surrounding the reviewed dilemmas of subclinical AF, secondary AF and post-stroke AF. Icons: a magnifying glass indicates definitions, a lightbulb indicates a key message from trials, tick-mark indicates recommendations. AF, atrial fibrillation, AHRE, atrial high-rate episode; CIED, cardiac implantable electronic device; ICH, intracerebral haemorrhage; OAC, oral anticoagulation; RCT, randomised controlled trial.

Finally, we include a section related to challenging decision-making following a recent stroke. This includes the timing of recommencement of OAC after ischaemic stroke or intracerebral haemorrhage (ICH), as well as advice for managing patients who have a ‘breakthrough’ stroke despite OAC therapy. A visual summary of the reviewed topics is presented in Figure 1.

## Subclinical AF and atrial high-rate episodes

### Nomenclature

Diagnosis of AF on a standard 12-lead ECG continues to be recommended as the gold standard; however, difficulty obtaining recordings during symptomatic periods for patients with paroxysmal arrhythmias remains a challenge.^2^ Alternative means of assessing the cardiac rhythm have historically included cardiac implantable electronic devices (CIEDs) such as pacemakers and implantable cardioverter defibrillators (ICDs), plus implantable cardiac monitors (ICMs) which may be implanted for arrhythmia diagnosis. More recently, a proliferation of consumer-grade wearable technology capable of recording single or multi-lead ECG traces offers an additional route to a diagnosis of AF.^4^

This multiplicity has necessitated the creation of new diagnostic entities, and the variability of terminology used in the literature may be confusing. Clinical AF – demonstrated on a 12-lead ECG or alternatively at least 30 s of AF on a single lead ECG recording is generally distinguished from subclinical AF (SCAF) – asymptomatic episodes demonstrated on other recordings (e.g. Holter monitors, CIEDs, wearable monitors). Within SCAF, atrial high-rate episodes (AHREs) refer to arrhythmias detected only by CIEDs: defined by the European Heart Rhythm Association consensus as episodes with an atrial rate greater than 190 bpm.^5^ SCAF tends to progress to clinical AF, at a rate of around 10% per year.^6^

The majority of the published literature uses data from CIED patients and hence refers to AHREs and SCAF synonymously. It is possible that the findings can be generalised to SCAF diagnosed on direct-to-consumer wearables that serve the same purpose (extended ECG monitoring), though as yet large trials in this clinical setting are awaited.

### Frequency of SCAF

Asymptomatic AF is more frequent than symptomatic AF^7,8^ and is not benign – associated with higher mortality, likely because asymptomatic patients are more likely to be older and have a higher comorbidity burden.^9^ Due to the overlapping risk factor profile, SCAF is common amongst patients with indications for cardiac pacing. Gillis and Morck reported a 68% incidence of AF in patients who received a device for sinus node disease and a 37% incidence among patients with AV block. Also notably, the burden of AF increased throughout the study period (of approximately 2 years).^10^

The prospective ASSERT study followed 2580 patients aged above 64, without known clinical AF, who had recently undergone pacemaker or ICD implantation.^11^ At 3 months, SCAF was detectable in 10.1%, and SCAF was strongly associated with the development of clinical AF (hazard ratio (HR): 5.56, *p* < 0.001). Another retrospective study of patients with pacemakers for sinus node dysfunction found that AHRE (here defined as >220 bpm for >10 beats, patients only included with at least one AHRE >5 min) was similarly associated with clinical AF (HR: 5.93, *p* = 0.0001), as well as mortality.^12^

### Stroke risk in subclinical versus clinical AF

Numerous large studies have assessed the impact of AHREs on the risk of future stroke. The aforementioned ASSERT study reported an increase in the rate of stroke or systemic thromboembolism by a factor of 2.5, though the overall event rate was fairly low, with only 51 events in their population of 2580 – an annualised rate of 0.8% (the median CHADS_2_ score of 2.3 would predict a rate of >4% per year).^11^ Similarly, a 2018 meta-analysis of three studies described an annual stroke rate of 2.76%, with a mean CHADS_2_ score of 2.1.^13^ Lau et al. established that whilst SCAF increased stroke risk, the temporal relationship between recorded episodes of AF and subsequent stroke events is inconsistent.

More recently, two large randomised trials of direct oral anticoagulant (DOAC) medications in patients with CIED-detected SCAF have been published. NOAH-AFNET6 (2536 participants) and ARTESIA (4012) compared DOAC therapy (edoxaban or apixaban, respectively) with placebo or aspirin.^14,15^ NOAH-AFNET6 reported a stroke incidence of 0.9% per year in the edoxaban group and 1.1% in the placebo arm. Given the median CHA_2_DS_2_-VASc score of 4 in both groups – the expected annual stroke rate for a reference population with clinical AF would be 4.8%.^16^ Likewise, the stroke rates in ARTESIA were 0.78% in the apixaban arm and 1.24% in the aspirin arm, with an overall mean CHA_2_DS_2_-VASc score of 3.9.

In summary, whilst SCAF/AHREs are clinically meaningful with regard to ongoing stroke risk (with approximately a 2.5-fold increase), their effect is not as significant as a diagnosis of clinical AF when adjusting for other stroke risk factors.

### Effect of event duration on stroke risk in SCAF

Given the stroke risk is lower than expected for the SCAF population as a whole, investigators have attempted to further stratify by the duration of the detected episodes, to identify candidates who may benefit from anticoagulation.

Firstly, very short AHREs do not appear to be clinically meaningful. Data from the RATE registry of 5379 patients show that episodes of AF less than 15–20 s did not confer any increase in the stroke risk over a 2-year follow-up,^17^ a finding that was later corroborated by Mahajan et al.’s meta-analysis.^13^

Beyond this, the threshold of effect has been difficult to pinpoint, and there exists variability between different analyses. In ASSERT, event durations ⩾6 min were predictive of stroke (HR: 1.77, *p* = 0.047); however, when stratified into quartiles by duration, only the group with the longest durations (⩾17.72 h) demonstrated significantly higher event rates (HR: 4.89). The discrepancy may be partially explained, as described above, by the low event rates in the study (only 51 events). Importantly, increasing AHRE duration maintained its predictive value across the spectrum of baseline stroke risk (as assessed by CHADS_2_ score), with the highest event rates observed in CHADS_2_ scores of >2.^11^

Other studies report different thresholds: TRENDS, a 2009 prospective observational study found a doubling of thromboembolic risk in the group with AHREs above the median duration (5.5 h).^18^ Here thromboembolism was inclusive of transient ischaemic attack (TIA) in addition to stroke and systemic thromboembolism and total event rates were again low (40 events from a population of >2400 patients, with an annualised event rate of 1.2%). Contrastingly, Boriani and colleagues, in their analysis of pooled data totalling 10,016 patients, identified the highest risk at AHREs of 1 h duration, with slightly lower rates beyond this.^19^ Other meta-analysed data do not conclusively demonstrate any association at all between SCAF burden and stroke.^20^

In another study, AHREs >5 min were predictive of major adverse cardiovascular events – though it is not clear to what extent this was driven by stroke.^21^

The risk-enhancing effect of increasing AHRE duration appears to be synergistic with other stroke risk factors. Botto et al. examined the relationship between both AHRE duration and CHADS_2_ score with thromboembolic risk. Splitting their cohort into three groups by duration: <5 min, ⩾5 min but <24 h and ⩾24 h, they showed that at lower baseline stroke risk (CHADS_2_ of 1), only patients with AHRE durations above 24 h were at elevated risk (4% annually). Contrastingly, at CHADS_2_ of ⩾2, the same 4% risk was observed with all AHRE >5 min.^22^

Whilst there is significant variability in the reported effect thresholds, data generally demonstrate that increasing durations of AHRE/SCAF portend a higher stroke risk. All these studies are limited by the low event rates which is a major challenge in this patient population.

### Anticoagulation in SCAF

Given the observed increase in stroke rates in patients with SCAF, the natural question is whether a net clinical benefit could be obtained with anticoagulation. The argument for starting anticoagulant therapy, therefore, is stronger for patients with long durations of AHREs with additional stroke risk factors, especially when multiple factors are present. The 2020 ESC guidelines advocate consideration of anticoagulation in patients with event durations ⩾24 h, high CHA_2_DS_2_-VASc scores (⩾2 in males or ⩾3 in females), where there is no diagnostic doubt of AF on the examined device tracings, and when bleeding risks are judged to be low.^2^ Nevertheless, these guidelines noted a lack of data to make specific recommendations and called for large randomised trials in this arena.

Two trials, both published in late 2023, set out to answer this specific question. The first, NOAH-AFNET 6, recruited over 2500 patients aged ⩾65 years with relatively high predicted stroke risk (average CHA_2_DS_2_-VASc score of 4), randomising them 1:1 to the DOAC edoxaban or placebo. The mean duration of AHREs was 2.8 h. The trial was prematurely terminated due to higher rates of major bleeding in the edoxaban arm without any clinical benefit detected, possibly limiting its ability to detect a small benefit of edoxaban.^14^

Secondly, the ARTESIA trial randomised around 4000 patients to either apixaban or aspirin therapy. The majority of the included patients had AHREs lasting 6 min to 6 h, and the mean CHA_2_DS_2_-VASc score was 3.9. In contrast to NOAH-AFNET 6, this study did register a reduction in stroke risk in the apixaban arm (0.78% vs 1.24% per patient-year, HR: 0.63, *p* = 0.007); however, this came at the expense of a concurrent increase in major bleeding (1.71% vs 0.94%, HR: 1.80, *p* = 0.001).^15^ Furthermore, the difference in bleeding is likely to have been lessened by the choice of aspirin as the control arm, which is known to increase bleeding (around half of patients in NOAH-AFNET 6 were also taking aspirin).^23^

Despite these two studies being seemingly at odds with one another, a subsequent meta-analysis found the results to be consistent (I^2^ heterogeneity = 0%). McIntyre et al. reported that when taken together, DOAC therapy reduced the risk of ischaemic stroke by 32% and increased major bleeding by 62%.^24^

At first glance, the figures would suggest net clinical harm associated with anticoagulation in AHREs. However, the impact of the events may not be equivalent. As Svennberg points out in her editorial, DOAC therapy prevented severe strokes (likely avoiding high levels of disability) and caused mostly non-fatal bleeding.^25^ Indeed, in ARTESIA, there was no difference in fatal bleeding, and the vast majority of major bleeding events required supportive or conservative treatment only.

Taken together, these data would support a clinical practice similar to that recommended by contemporary guidelines, justifying the initiation of anticoagulant medications in patients with high CHA_2_DS_2_-VASc scores with relatively low bleeding risk, if observed durations of SCAF are longer.

## AF triggered by a significant stressful event

### Secondary AF – triggered or unmasked?

Commonly in clinical practice, the first diagnosis of AF is made incidentally during admission to secondary care for another reversible pathology, such as sepsis, surgery, thyrotoxicosis or acute pulmonary embolism.^26^ Clinicians often consider AF in this scenario to be a secondary phenomenon with a high chance of resolution after treatment of the precipitating condition, but long-term stroke risk is increased after first-diagnosed AF in the context of sepsis,^26,27^ non-cardiac^28^ and cardiac surgery.^29^

These data would imply an alternative framing of the situation – that patients with a predisposition towards AF (or pre-existing SCAF) have their condition unmasked by the stimulus and would likely have later developed the condition regardless.

Nevertheless, the evidence base for anticoagulation decision-making for this patient group is not robust, and this is reflected in the wide variation in clinical practice for in-hospital and post-discharge anticoagulation initiation. A UK-wide survey of predominantly intensive care physicians indicated that around two-thirds do not routinely anticoagulate critically ill patients with new-onset AF, whereas one-third anticoagulate within 72 h.^30^

Of the secondary triggers, the commonest with the largest evidence base are sepsis and surgical procedures which are reviewed below.

### New-onset AF associated with sepsis

Infection is one of the most common triggers of new-onset AF, accounting for over 20% of cases associated with a secondary precipitant.^26^ Pathophysiologic hypotheses for the association include electrolyte derangement, atrial stretch due to dysregulated volume status and increased myocyte automaticity in the setting of severe inflammation.^31^ Indeed, the degree of inflammation, as measured by serum C-reactive protein, is correlated with the risk of developing AF during infection.^32^

Inflammation induces a hypercoagulable state^33^; thus, it is unsurprising that new-onset AF during sepsis is associated with higher rates of in-hospital thromboembolic events, including stroke. Walkey et al., in their retrospective analysis of almost 50,000 patients with severe sepsis, found that new-onset AF increased the likelihood of in-hospital stroke to 2.7% compared with 0.6% in patients without AF, as well as in-hospital mortality.^34^ However, initiation of anticoagulation during the index admission is not without risk: two studies have shown higher rates of bleeding complications in the short term.^35,36^

Studies that examine longer-term risks associated with sepsis-driven AF suggest that the deleterious effects persist many years after hospital discharge. Firstly, new-onset AF during admission for infection increases the likelihood of subsequent AF. Reported effect size varies with HRs as high as 26.0 in one Danish registry study^37^ and 5.4 in another analysis on US Medicare recipients.^27^ The latter also noted a 54.9% rate of post-discharge AF diagnosis at 5 years, correlating with findings from the Framingham Heart Study (5-, 10- and 15-year recurrence rates for secondary AF of 42%, 56% and 62%, respectively).

Additionally, rates of ischaemic stroke and other thromboembolic events remain high following sepsis complicated by AF. Gundlund et al. described a doubling in long-term thromboembolic risk in sepsis survivors with AF compared to patients with sepsis but without AF,^37^ whereas Walkey et al. showed a 22% increased risk of ischaemic stroke at 5-year follow-up.^27^ A retrospective chart review of patients in Taiwan found new-onset AF during septicaemia to increase the stroke risk at a mean 4.6-year follow-up (HR: 1.88, 95% CI: 1.33–2.65).^38^ Finally, a meta-analysis of 12 studies performed by Arero et al. found new-onset AF to be a significant risk factor for stroke following sepsis hospitalisation (adjusted HR: 1.80, 95% CI: 1.42–2.28).^39^

We have seen that around half of cases of sepsis-driven AF recur and that long-term outcomes, especially stroke, are poor. However, all the aforementioned studies are observational and there is, to date, no evidence from randomised trials for the use of OAC in sepsis-driven AF. Ultimately, this leads to the perception of sepsis-driven AF as a marker of disease severity rather than a pathogenic mediator. Furthermore, there are likely to be short-term net harms from early anticoagulation during sepsis. As such, there is no clear guidance issued by major societies for this patient group.

A reasonable strategy is set out by Induruwa et al. in their review.^31^ Individuals should undergo stroke risk assessment via the CHA_2_DS_2_-VASc score as well as an assessment of bleeding risk factors. Where there are additional stroke risk factors, patients should in most cases be offered anticoagulation following a frank discussion of the risks and benefits.^40^ In borderline cases, extended cardiac monitoring is valuable given the high chance of recurrence.

Moreover, given the paucity of data for anticoagulation, the authors suggest a greater focus on cardiovascular risk factor management – as described in the Atrial Fibrillation Better Care (ABC) pathway, which represents the current holistic or integrated care approach to AF management that has been associated with improved clinical outcomes.^41,42^ This is important given the clinical complexity associated with AF, as these patients are often frail and have multimorbidity and pharmacy, with implications for treatments and clinical outcomes.^41,43,44^

Consistently, a 2023 consensus statement by the American Heart Association advocates a higher than usual threshold for anticoagulation initiation following acute AF (a term substituted by the authors for secondary AF) – with CHA_2_DS_2_-VASc ⩾2 for men and ⩾3 for women.^45^ This is reflective of the trajectory of the field – recognition of sepsis-driven AF as an important clinical entity but acknowledgement that randomised trial data are so far insufficient to justify more aggressive anticoagulation strategies.

### Post-operative AF

Surgery and the peri-operative period involve a major physiologic stress and frequently it is complicated by new-onset post-operative AF (POAF). Typically POAF occurs between post-operative days 2 and 4^46^ and is particularly common following cardiac surgery, which involves manipulation and frequent cannulation of atrial anatomic structures.

Previously, incidence has been reported as 30%–50% for cardiac surgery (with variation depending on the type of operation)^46,47^ and 5%–10% for major non-cardiac surgery.^48^ Recent studies report more conservative figures, with incidences of 18.8% for cardiac surgery and 0.8% for non-cardiac surgery,^49^ which may reflect increased use of prophylactic measures as described in contemporary guidelines, or equally under-detection of shorter, asymptomatic episodes.^2^

### POAF following cardiac surgery

AF is the most common complication of cardiac surgical procedures and frequently presents acute challenges involving haemodynamic instability and heart failure.^46^ Though incompletely understood, putative contributory factors include inflammation of the pericardium, intraoperative volume shifts and alterations in the neurohormonal milieu post-operatively.

POAF is strongly associated with myriad post-operative complications, such as reoperation, development of sepsis, need for prolonged ventilatory weaning or dialysis and mortality. It is unclear whether this represents a different risk profile or whether AF plays a causal role in these negative outcomes.^50^

The relationship with ischaemic stroke, however, is less clear, and it appears that POAF does not carry the same long-term stroke risk as non-surgical AF.^51^ Estimates of the effect size vary significantly. A meta-analysis of four studies including approximately 9000 patients reported a four-fold increase in the risk of ischaemic stroke associated with POAF.^52^

In a large cohort study of over 1,700,000 patients, Gialdini et al. found a more modest increase in stroke risk (HR: 1.3, 95% CI: 1.1–1.6) with relatively low overall rates (0.99% in the AF group), corresponding with findings from Lin et al. who demonstrated a 20% increased risk.^53,54^ Contrastingly, Bianco et al. found no significant difference in stroke (2.76% vs 2.32% with and without POAF, respectively, *p* = 0.191). The explanation for the variability is uncertain, but it seems likely that at least a small difference does exist, and some investigators believe this reflects a subset of POAF patients who may more closely resemble ‘non-operative AF’ and benefit from conventional anticoagulation strategies.

On the flip side, anticoagulation is a high-risk intervention in this patient group, particularly if initiated early. It is associated with an increased risk of significant complications like cardiac tamponade, as well as other bleeding events.^55^ Additionally, the efficacy of OAC in reducing stroke has not been demonstrated. A large observational study of OAC for POAF following coronary artery bypass grafting noted an associated increase in bleeding (HR: 1.60, 95% CI: 1.38–1.85) and mortality (HR: 1.16, 95% CI: 1.06–1.26) without any accompanying reduction in stroke rates (HR: 0.97, 95% CI: 0.82–1.15), even in patients with high CHA_2_DS_2_-VASc scores (>5).^56^ These findings are limited by the observational nature of the study and are hence subject to bias – given the lack of evidence, clinician behaviour may lead OAC medications to be commenced only in patients with very perceived risks, who are likely to be older and more comorbid.

The 2020 ESC guidelines recommend that OAC may be considered for POAF after cardiac surgery, after considering the stroke and bleeding risk profiles of the patient (IIb recommendation, level of evidence B). In practice, given the lack of randomised trials, a conservative approach is common, reserving anticoagulation for patients with a significant expected net benefit.^2^

### POAF following non-cardiac surgery:

Non-cardiac operations are also frequently complicated by new-onset AF, though not as commonly as after cardiac surgery.^49^ The difference may be explained by a lower burden of cardiovascular comorbidities, as well as less direct impact of surgery on atrial myocardium. Proposed arrhythmic triggers in the peri-operative period include volume loss, inflammatory cytokine release and high concentrations of circulating catecholamines.^45^ POAF is typically short-lived and around 40% of cases revert to sinus rhythm without any treatment within the admission,^57^ but similarly to other forms of secondary AF there is increasing recognition that the long-term trajectory is not benign, associated with incident heart failure, myocardial infarction, stroke and mortality.^49,58^

The largest synthesis in this area has been carried out by Lin et al.; in their 2019 meta-analysis totalling 2.5 million patients, the authors found POAF after non-cardiac surgery to be more strongly associated with long-term stroke risk (HR: 2.0, 95% CI: 1.70–2.35) than following cardiac surgery (HR: 1.2, 95% CI: 1.07–1.34). The only recent data not included in this analysis come from a cohort study by Butt et al.^59^ The authors found comparable stroke risk with POAF and non-surgical AF around 3% risk per year, though the high event rate and very low incidence of POAF (0.4%) relative to other studies are notable, and may suggest under-detection of short-lived, lower-risk cases.

As to whether anticoagulation is of benefit to these patients, there are very limited data to guide decision-making. A relatively small meta-analysis of 29,566 patients with POAF related to non-cardiac surgery was recently published by Neves et al.^60^ The key findings were that anticoagulation use was associated with a non-significant reduction in thromboembolic risk (odds ratio (OR): 0.71, 95% CI: 0.33–1.15) but a significant increase in bleeding (OR: 1.2, 95% CI: 1.10–1.32), and no effect on mortality. It is very difficult to make robust inferences from observational data, and there is a need for randomised trials of this patient group.

As seen with the examples of ‘secondary AF’ thus far, there is a perceived spectrum of intensity for any particular triggering stimulus for AF. The more intense the stressor, the less likely that the AF is a result of pre-existing arrhythmogenic substrate and so lesser is the long-term-associated risk. Cardiac surgery seems to exist at one end of the spectrum such that the outcome profile of POAF following cardiac procedures does not seem to resemble that of non-secondary AF.

On the other hand, POAF after non-cardiac operations appears to confer long-term risks more similar to those of non-secondary AF. However, history tells us that many logical concepts do not survive first contact with a randomised trial, and inferring a net benefit of OAC to negate the stroke risks in these populations is risky without such data. Currently, guidelines recommend that OAC *should be considered* for POAF after non-cardiac surgery, after considering the stroke and bleeding risk profiles of the patient (IIb recommendation, level of evidence B).^2^

## Anticoagulation conundrums in patients with recent stroke

### Anticoagulation timing after acute ischaemic stroke in patients with AF

The optimal timing for (re-)initiating OAC following an acute ischaemic stroke in patients with AF remains uncertain, and balancing the risk of recurrent stroke against the competing risk of secondary haemorrhagic transformation is crucial. In the study by Chang et al., patients with severe stroke were at higher risk of haemorrhagic transformation following early OAC initiation compared with late, whereas no increased risk was observed in patients with mild-moderate stroke.^61^

Current European guidelines recommend the (re-)initiation of OAC 1–3 days after a TIA, depending on brain imaging findings, or at ⩾3, ⩾6–8 or ⩾12–14 days after a mild, moderate or severe ischaemic stroke, respectively, in the absence of haemorrhagic transformation.^62^

The Early Versus Delayed Non-Vitamin K Antagonist Oral Anticoagulant Therapy After Acute Ischaemic Stroke in Atrial Fibrillation (TIMING) trial assessed the noninferiority of early versus late initiation of DOACs.^63^ Patients with acute stroke were randomised within 72 h of symptom onset to early (⩽4 days) or delayed (5–10 days) DOAC initiation. The primary outcome – a composite of recurrent ischaemic stroke, symptomatic ICH or all-cause death within 90 days – showed early initiation was non-inferior to delayed initiation. Notably, early initiation was associated with numerically lower rates of ischaemic stroke and death, with no symptomatic ICH recorded in either group during the 90-day follow-up. However, the study was underpowered and did not stratify patients according to stroke lesion severity.

The Early versus Late Initiation of Direct Oral Anticoagulants in Post-ischaemic Stroke Patients with Atrial Fibrillation (ELAN) trial, an international multicentre study, compared early (within 48 h after a minor or moderate stroke or on days 6 or 7 after a major stroke) versus late (days 3 or 4 after a minor stroke, days 6 or 7 after a moderate stroke or days 12, 13 or 14 after a major stroke) initiation of DOAC in patients with AF.^64^ No statistically significant difference was observed between the groups regarding the primary outcome – a composite of recurrent ischaemic stroke, systemic embolism, major extracranial bleeding, symptomatic ICH or vascular death within 30 days after randomisation. Similarly, no difference was found in the risk of recurrent ischaemic stroke, ICH or vascular death at 30 and 90 days. These data suggest that early DOAC use after ischaemic stroke does not increase harm, potentially indicating an important shift in clinical practice and guidelines.

The Optimal Timing of Anticoagulation after Acute Ischemic Stroke (OPTIMAS) trial, registered on ClinicalTrials.gov (NCT03759938), is underway to provide more evidence on the topic above.

### Anticoagulation decision-making in patients with AF and recent ICH

The evidence base to support OAC decision-making for ischaemic stroke prevention in patients with AF and recent ICH is limited. One meta-analysis published by Ivany et al. indicated that OAC effectively reduces the risk of ischaemic stroke in patients with AF post-ICH (relative risk (RR): 0.51, 95% CI: 0.30–0.86, *p* = 0.01), without significant elevating the risk of recurrent ICH (RR: 1.44, 95% CI: 0.38–5.46, *p* = 0.59).^65^ However, the studies underlying these findings exhibit methodological heterogeneity, including differences in participant inclusion/exclusion criteria, follow-up durations and types of OAC utilised.

The principal obstacle to OAC therapy for stroke prevention in patients with AF and ICH is the concern and uncertainty over major bleeding risks.^66^ A survey among clinicians revealed that fears of recurrent ICH were the predominant reason for hesitance to initiate or resume OAC in patients with AF post-ICH, with 63% of clinicians expressing apprehension about major bleeding.^67,68^ This anxiety is partially mirrored by patients with AF who have recently experienced ICH.^69^

This variability in clinical practice regarding OAC use for ischaemic stroke prevention in patients with AF post-ICH primarily stems from the scarcity of robust evidence on the topic.^67^

### Breakthrough ischaemic stroke in patients with AF already on oral anticoagulation

Despite advancements in stroke prevention, patients with AF maintain an annual ischaemic stroke risk of 1%–3% even when on effective OAC treatment.^70^ A pooled analysis of seven prospective cohort studies revealed that AF patients experiencing stroke while on OAC had a higher risk of stroke recurrence than OAC-naïve patients, despite comparable CHA_2_DS_2_-VASc and HAS-BLED scores.^71^ The authors also found no benefit to changing the OAC type post-index event on the risk of subsequent strokes. This highlights the challenge of managing breakthrough strokes, where the optimal antithrombotic strategy remains uncertain.

Ip et al. investigated antithrombotic strategies for AF patients on DOACs at the time of an ischaemic stroke.^72^ The study evaluated four strategies: continuation of the same DOAC (DOAC-same), switching from DOAC-to-warfarin, switching between DOACs (DOAC-switch) and adding antiplatelet agents. The study found that continuing the same DOAC was associated with the lowest annual risk of recurrent stroke (8.7%). By contrast, both DOAC-switch and DOAC-to-warfarin switch strategies were linked to a higher risk of recurrent stroke compared to the DOAC-same strategy (adjusted hazard ratio (aHR): 1.96, 95% CI: 1.29–3.02, *p* = 0.002, and aHR: 1.62, 95% CI: 1.25–2.11, *p* < 0.001, respectively). Adding antiplatelet treatment to the DOAC-same strategy did not significantly reduce the risk of recurrent ischaemic stroke (aHR: 1.28, 95% CI: 0.88–1.84, *p* = 0.188), ICH (aHR: 1.20, 95% CI: 0.54–2.68, *p* = 0.654) or death (aHR: 1.09, 95% CI: 0.84–1.41, *p* = 0.512). Furthermore, there were no significant differences in the risk of ICH and death among the groups.

These data indicate that continuation of the same OAC is preferable following a breakthrough ischaemic stroke.

## Conclusion

AF is a very diverse condition with a wide spectrum of associated risks. The benefits of OAC in routine practice for patients with AF are well established; however, many grey areas exist where estimating the balance of risk and benefit is challenging. We have reviewed short runs of SCAF (detected by CIEDs or wearable monitors), AF secondary to sepsis or the peri-operative period, as well as common dilemmas relating to recent stroke or ICH.

SCAF carries a modestly enhanced risk of stroke when compared with clinical AF, and the benefits of anticoagulation are smaller. A tailored approach is therefore required – with OAC reserved for patients with longer episodes (approaching 24 h), with higher CHA_2_DS_2_-VASc scores, and lower estimated bleeding risk.

AF triggered by sepsis recurs in over 50% of patients and carries a greater risk of ischaemic stroke. The consensus opinion is that patients with particularly high stroke risk (CHA_2_DS_2_-VASc ⩾2 for men and ⩾3 for women) should warrant consideration of anticoagulation.

Patients with POAF seem to derive less benefit from anticoagulation, despite the increase in stroke. Data supporting the routine use of OAC is currently lacking, and guidelines advocate consideration of OAC initiation only if the balance of risks is clearly favourable. Cardiac surgery-related POAF appears to be a special case – it is induced very frequently and the associated risks appear to be much lower. OAC use in this population is rarely indicated.

The evidence base for secondary AF is plagued by a lack of randomised clinical trials and therefore subject to possible bias and confounding. Higher-risk interventions like OAC should therefore have a higher threshold for initiation, with more focus on other aspects of the holistic ‘ABC’ care pathway as set out in the European guidelines.

Recent evidence suggests that early (re-)initiation of OAC for secondary prevention in patients with AF does not increase the risk of recurrent ischaemic stroke or ICH. Breakthrough strokes in patients with AF who were already on DOAC pose an important clinical dilemma, due to multiple potential competing stroke aetiologies, but changing to an alternative OAC appears to be deleterious. There is a paucity of evidence to guide the decision for OAC in patients with AF and recent ICH.

## Declarations

**Ethics approval and consent to participate:** Not applicable.

**Consent for publication:** Not applicable.

**Availability of data and materials:** No new data were generated by, nor was the original data of any study analysed in this work.
